# Older Adults’ and Professionals’ Attitudes Towards Stair-Fall Prevention Interventions

**DOI:** 10.3390/healthcare13111324

**Published:** 2025-06-02

**Authors:** Emma Mulliner, Thomas D. O’Brien, Vida Maliene, Constantinos N. Maganaris, Rachel Mason

**Affiliations:** 1 Faculty of Health, Innovation, Technology and Science, Built Environment and Sustainable Technologies Research Institute, Liverpool John Moores University, Liverpool L3 3AF, UK; v.maliene@ljmu.ac.uk; 2 School of Sport & Exercise Sciences, Faculty of Health, Innovation, Technology and Science, Liverpool John Moores University, Liverpool L3 3AF, UK; t.d.obrien@ljmu.ac.uk (T.D.O.); c.maganaris@ljmu.ac.uk (C.N.M.); 3 Department of Land Use Planning and Geomatics, Faculty of Engineering, Vytautas Magnus University Agriculture Academy, 53361 Akademija, Lithuania; 4 Medical School, Faculty of Health & Medicine, Lancaster University, Lancaster LA1 4AT, UK; r.mason5@lancaster.ac.uk

**Keywords:** ageing, ageing in place, fall prevention, older adults, stairs, stair falls, stakeholder engagement, stakeholder attitudes

## Abstract

**Background/Objective:** Stair falls are a major health concern for older adults, particularly those wishing to age in place. Despite extensive laboratory research on the causes of stair falls and the effectiveness of prevention interventions, there is limited understanding of how acceptable interventions are to end-users and key stakeholders in real-world home environments. This study explored older adults’ and professionals’ attitudes toward stair-fall prevention interventions, including intervention acceptability, barriers and facilitators to adoption, and priorities for implementation in home settings. **Methods:** This study employed a sequential mixed-method design, including a survey of 359 UK community-dwelling older adults (aged 55+), followed by focus groups with 8 older adults and 11 professionals from healthcare and housing backgrounds. **Results:** Older adults surveyed perceived home stair falls as a significant risk and priority for prevention but demonstrated less awareness of specific interventions to prevent falls. Focus groups with older adults and professionals established barriers and facilitators to the adoption of 10 specific stair-fall prevention interventions. Barriers included a lack of awareness, financial constraints, reluctance to alter home environments and stigma. Facilitators included raising awareness through education, clear guidance on intervention benefits and installation, practical and financial support, personalised approaches, social encouragement, and endorsement by professionals. Focus groups found the most acceptable stair-fall prevention interventions included education and skill training, improved staircase lighting and additional handrails. **Conclusions:** Interventions that are low-disruption, cost-effective, backed by empirical evidence, and endorsed by trusted professionals are more likely to be accepted and implemented. Further research should focus on targeted educational strategies to overcome barriers to adoption.

## 1. Introduction

Falls are increasingly common, and the resulting injuries are recognised as a problem worldwide [1,2]. This presents a serious concern for older adults, given that a third of people aged over 65 and half of those over 80 fall each year [3,4]. In the UK, falls are the most common cause of fatal and non-fatal accidents involving people over the age of 65 years, and the leading cause of death from injury among people aged over 75 years [5]. As well as devastating human costs, falls are estimated to cost the National Health Service (NHS) and social care GBP 6 m per day or GBP 2.3 bn per year [6]. With global population ageing, the problem is predicted to escalate and further strain already over-burdened healthcare systems [7]. Therefore, finding effective methods to prevent falls in older adults is a critical global challenge and public health priority [1,8,9]. Most older adults’ falls occur indoors [4,9]. Moreover, over 90% of people aged 65 years and over in the UK live in mainstream housing [10], with the majority desiring to ‘age in place’ [11]. Therefore, fall prevention within the home environment is crucial to support independent healthy ageing [9].

Stair use is one of the most demanding and hazardous activities for older adults and a common cause of falls [12,13,14]. Stair falls are especially concerning because they are associated with the most severe injuries, are a leading cause of death among older adults, and result in high economic cost [12,14,15]. The Royal Society for the Prevention of Accidents reports that over 60% of accident-related deaths amongst the elderly in the UK are due to falls involving stairs or steps, and the home is the most common location for stair falls [16]. Thus, it is crucial that interventions to prevent stair falls are implemented in older adults’ homes. The design of stairs and the surrounding environment is intuitively the first factor to consider. National building regulations—approved Document K—currently exist that specify the physical characteristics of stairs in new dwellings [17]. However, the UK possesses one of the oldest housing stocks in Europe [18], with stairs in older homes likely deviating from these modern safety standards. In addition to improving stair design and the surrounding environment [19,20,21,22], there is a growing body of knowledge documenting that stair-fall risk may be ameliorated by interventions targeting individual functional capability deficits [23,24,25,26,27]. However, this knowledge comes from controlled studies in lab environments and has not been implemented in real home settings. To successfully translate lab-based stair safety research into practice, we must understand stakeholder perceptions about the acceptability of these interventions.

The complexity and importance of establishing stakeholder engagement in fall prevention have been documented in the literature on general (non-stair-specific) fall prevention, which highlights that the translation and implementation of research into policy and practice are problematic [3,27,28]. Whilst effective fall prevention interventions have been identified in randomised controlled trials, fall rates in older adults have not reduced accordingly [29,30]. Reasons limiting fall reduction efforts are said to include the insufficient understanding of stakeholder (including older people, healthcare, and related professionals) views of fall prevention [31,32] and challenges persuading older adults to adopt interventions [8]. Optimal fall prevention requires person-centred approaches and shared decision making, including the consideration of attitudes and preferences of older adults and perspectives from other stakeholders [1,9,28]. Investigating stakeholder perceptions has been identified as a way to enhance the translation of knowledge [30,33]. Research examining older adults’ attitudes [3,8,31,34,35,36,37,38] and professional stakeholders’ attitudes [30] towards general fall prevention and related interventions is developing. However, such studies do not focus on stair falls specifically. Given the unique challenges of stair negotiation and the specialised interventions, which may include modifications to a person’s home, it is vital that the views of both older adults and professional stakeholders towards stair-fall prevention interventions are established. Currently, such research is limited. While Tural et al. [14] investigated older adults’ attitudes toward stair-fall prevention interventions in homes, the study focused solely on stair mobility products. Moreover, little is known about how the attitudes of older adults triangulate with those of professional stakeholders, which are important to consider since they impact the endorsement and the practical implementation of interventions and are key in shaping related policy concerning stair falls and safety. To address this gap in the literature, the purpose of this study is to understand older adults’ and professional stakeholders’ attitudes towards stair-fall prevention interventions, including interventions that have previously been shown to be effective for reducing stair-fall risk [19,20,21,22,23,24,25,26], in order to identify barriers and facilitators to their successful adoption and implementation in real home and community settings.

## 2. Materials and Methods

This study adopted a sequential mixed-method design commencing with quantitative data collected through a survey, followed by focus groups to explore the results and topic at a deeper level [39]. Incorporating both approaches accommodates strengths in research approach and outcomes [40,41].

### 2.1. Survey

A survey of older adults aimed to understand general attitudes toward stair-fall risk and awareness of stair-fall prevention. Inclusion criteria were UK residents aged 55 and over living in mainstream (community dwelling) housing containing stairs. The survey questionnaire was developed by the research team to meet the specific aims of this study (see Appendix A). The survey contained questions on the following: participant demographics; property characteristics and living arrangements; awareness of stair falls and risk factors; personal perception of fall risk; awareness of and interest in stair-fall prevention. Questions were evaluated using multiple-choice lists and Likert statements.

The survey was created and distributed primarily using JISC online platform. A pilot survey was tested with academics aged 55+ at the researchers’ institution. The link to the online survey was distributed to the general public and professional networks via social media (LinkedIn, Facebook, and X (previous Twitter)) and shared through relevant older adult networks and charities (e.g., AgeUK). Additionally, flyers with a link to the online survey and physical paper copies of the survey were left in public places (e.g., health services, pharmacies, and community centres) and distributed in forums held with older adults in the local area of the researchers (Merseyside, England). Similar to other studies involving older adults [14,42,43,44], primarily online administration was selected to increase geographical reach and response rates, as well as offering participants anonymity and convenience (allowing respondents to complete the survey at their own pace, in a familiar environment). The prevalence of daily internet use among adults aged 50+ in England reached 74% in 2020 [45]. However, it is acknowledged that the online survey method may exclude some digitally disconnected individuals, potentially introducing sample bias.

Based on UK older adult population estimates [46], a target sample size of 385 participants was calculated to achieve a 95% confidence level with a 5% margin of error. The final valid sample size achieved was 359, which is near this benchmark and exceeds the minimum required for a 90% confidence level with a 5% margin of error (n = 271) and a 95% confidence level with a 6% margin of error (n = 267). This sample size was considered sufficient to draw reasonably representative conclusions about the broader older adult population in the UK. Descriptive analysis was carried out on the survey data, and findings were used to inform the next phase of data collection.

### 2.2. Focus Groups

Two face-to-face focus groups were conducted, one with older adults and one with professional stakeholders. The first focus group included eight older adults aged 60–84, including both fallers (had previously experienced a stair fall) and non-fallers (had not previously fallen on stairs). Participants were recruited from survey respondents who expressed interest in participating in further research. A second focus group was held with 11 professionals from health and housing disciplines. Professionals were purposively selected from health and housing sectors, with direct involvement in stair falls in homes and/or issues with stair safety.

Findings from the preceding survey helped inform the development of semi-structured topic guides for the focus groups, which aimed to explore attitudes toward specific stair-fall prevention interventions. A total of ten interventions were discussed in both focus groups, including interventions involving changes to the home environment, to physical capabilities of the individual, and to personal behaviour (see Table A1, Appendix B). These interventions were selected based on the survey results and supported by previous published evidence indicating their effectiveness in ameliorating stair-related fall risk specifically [19,20,21,22,23,24,25,26] and fall risk in general [47,48]. Separate focus groups were conducted with older adults and with professionals. The older adult focus group examined participants’ perceptions of the acceptability of each intervention, as well as perceived barriers and facilitators to their adoption in real-life home environments. The professional focus group followed a similar structure but concentrated on the practical feasibility of implementing each intervention, alongside identifying implementation priorities and informing directions for future research. Both focus groups concluded by employing a consensus-building approach. Among older adults, consensus was sought on the perceived acceptability of each intervention using a four-point scale: not acceptable, probably not acceptable, probably acceptable, and acceptable. Among professionals, consensus focused on implementation priority, using the following four-point scale: not a priority, low priority, moderate priority, and high priority. The discussions lasted around 120 min (with older adults) to 150 min (with professionals), including breaks. Researchers took notes, and the discussions were recorded and transcribed.

Thematic analysis was conducted using an inductive, realist approach, assuming that the interviews accurately captured participants’ experiences, meanings, and realities. Patterns and ideas were identified, coded, and organised into key themes [49]. These themes were further refined through iterative discussions within the research team.

## 3. Results

### 3.1. Survey Results

The characteristics of the 359 older adult survey participants can be found in Table 1.

The majority (92%) of participants agree that stair falls are a concern for older adults, and 85% agree that stair falls are a common occurrence for older adults. However, fewer participants (67%) considered themselves personally at risk of falling on stairs, with 42% perceiving themselves as ‘fit and healthy, thus, not at risk of stair falls’.

**Figure 1:**
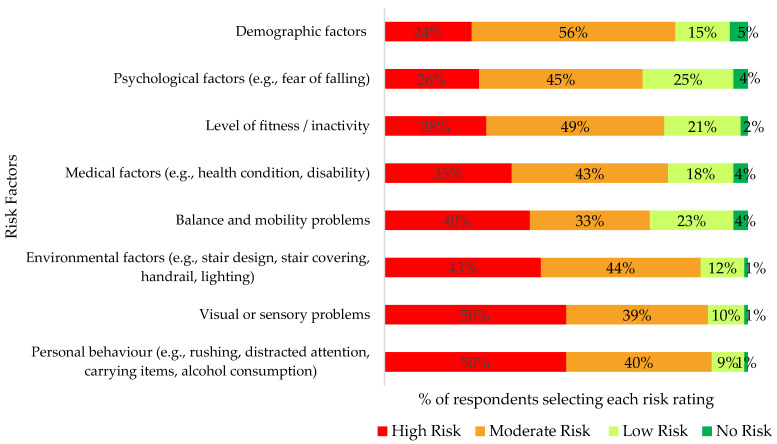
Ranking of risk factors for stair falls by older adult respondents.

Over half (56%) of participants had experienced a stair fall, with 63% of falls occurring on stair descent. Almost half (47%) of survey participants who had experienced a stair fall cited their own behaviour as the cause of the fall, while 28% attributed falls to the staircase environment, 19% due to health status or a medical condition, and 6% due to a combination of other factors. All older adult participants (previous fallers and non-fallers) were asked to rank risk factors for stair falls (as either high, moderate, low, or no risk). The top three perceived risk factors included ‘personal behaviour’, ‘visual or sensory problems’, and ‘environmental factors’ (Figure 1).

Over a third (35%) of participants thought their own stairs were unsafe or presented a fall risk. Reasons cited for stairs being unsafe included the following: step dimensions or steepness (17%); narrow staircase (14%); poor lighting (16%); handrail disrepair (13%), inappropriate height (10%) or missing step (7%); type of stair covering (13%); stair décor, e.g., busy carpet pattern (7%); and cluttered environment (5%).

**Figure 2:**
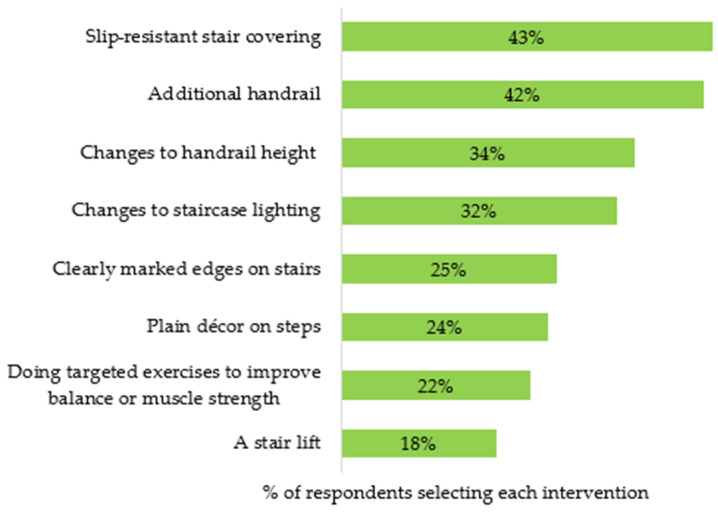
Older adults’ perception of good options for stair-fall prevention intervention.

Most participants agreed that stair falls are preventable (89%) and that home stair-fall prevention is a priority (81%). A total of 83% of participants suggested that they were either ‘interested’ (46%) or ‘possibly interested’ (37%) in making changes to the staircase environment to prevent falls. Similarly, 83% of participants were ‘interested’ (48%) or ‘possibly interested’ (35%) in making behavioural changes to prevent stair falls. Participants were asked to select specific stair-fall prevention interventions that they perceived would be a good option to improve home stair safety (Figure 2), with the most common selections including changes to stair coverings and handrails. While 83% of participants indicated some interest in behaviour interventions to prevent falls and 73% perceived ‘balance and mobility problems’ to be a moderate or high-risk factor for stair falls (Figure 1), only 22% selected targeted physical exercise as a good option (Figure 2).

The most common barriers to adopting interventions to prevent home stair falls were found to include ‘not knowing where to get reliable advice’ (35%) and ‘affordability/cost’ (31%). Other barriers included ‘not knowing what intervention might be possible’ (27%), ‘needing assistance to make adaptions/changes’ (21%), ‘concern about appearance of physical changes to home’ (20%), ‘thinking they are not needed yet’ (19%), ‘other people in the home not wanting them’ (16%), and ‘stigma’ (8%). The most common facilitators to the adoption of interventions included ‘better understanding of benefits of stair-fall prevention’ (53%) and ‘better understanding of intervention options’ (49%). Other facilitators included ‘hearing about interventions from others who have used them’ (31%), ‘being referred by a healthcare professional’ (27%), and ‘social support’ (24%).

These survey results provided an umbrella view of older adults’ opinions on home stair-fall risks and stair-fall prevention. While the survey identified broad barriers and facilitators to the adoption of stair-fall prevention interventions, the relevance to specific interventions required investigating in more depth. The survey results informed the design of the focus group discussions that allowed deeper exploration of stakeholder attitudes towards specific interventions, to gain understanding of why specific interventions may be acceptable or unacceptable for adoption and implementation by both end-users and professional stakeholders.

### 3.2. Focus Group Results

#### 3.2.1. Focus Group Participants

The first focus group was with eight older adults aged 60–84 participating in a face-to-face focus group setting. All participants lived in mainstream housing containing stairs in the UK. Further characteristics of the participants and their home setting are summarised in Table 2. The second face-to-face focus group involved 11 professional stakeholders from health and housing backgrounds. Participant information can be found in Table 3.

#### 3.2.2. Attitudes Towards Stair-Fall Prevention Interventions

**Figure 3:**
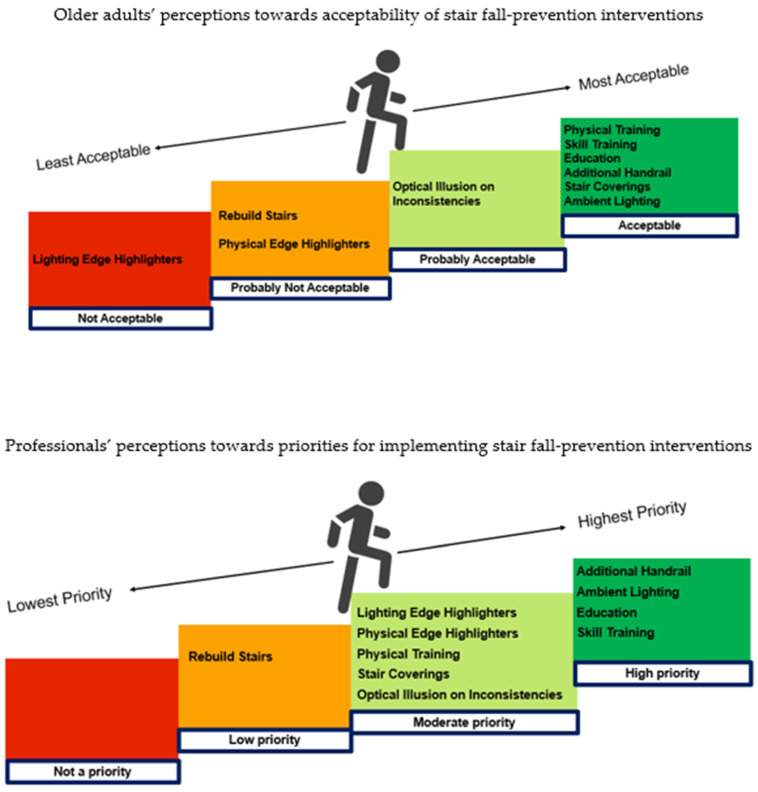
Summary of older adults’ consensus ratings compared to professionals’ consensus ratings towards stair-fall prevention interventions.

Focus group results are presented in this section, intervention by intervention (as listed in Table A1, Appendix B), with older adult attitudes preceding professional attitudes. A summary of the main themes (Table 4) and consensus (Figure 3) emerging from the focus group discussions are provided to conclude this section.

1. Rebuilding stairs: All older adult participants identified practical concerns as a barrier to rebuilding stairs; P8 stated, “*Apart from the cost for many older people, the sheer upheaval and working out how you find the responsible tradesmen to do the job properly are problems*”; including space constraints; P2 stated, “*In terraced houses the space is very confined, so you’re not going to be able to make a longer or wider staircase*”. For older adults living with others or renting, having independent decision-making power or control to make significant adaptions at home was identified as a barrier. Overall, there was low acceptability of rebuilding stairs as an intervention with consensus from older adults that “*the negatives would outweigh the positives*” (P3), unless it was essential. Participants suggested that safe stair dimensions should be a key consideration in new build construction and consideration by older adults if moving home.

Comparable with older adults, professionals identified cost and space constraints as key barriers to implementing this intervention. Housing professionals emphasised that changing stair dimensions can require knocking down walls and taking space out of other rooms in the home, which may be undesirable or prohibitively expensive. S2 cautioned: “*The truth is you’re going to have to rehouse someone whilst you’re doing this work, [……] it’s not really that viable unless they’ve got some very obliging neighbours or friends or family that they can go and stay with*”. There was consensus among professionals that rebuilding stairs would be a low priority and more cost-effective environmental interventions should be considered first.

2. Additional handrail: Older adults agreed that a handrail is pivotal for stair safety, providing a sense of security and stability. “*I wouldn’t go up the stairs unless I was holding onto the banister, I would never go up without holding on to something*” (P4). Handrails were seen as a way of assisting with balance problems that progressively worsen with ageing and providing confidence when using stairs. Only one of the participants already had two handrails on their home staircase, but most participants acknowledged that they would feel safer using stairs if they had an extra handrail (or two). However, space constraints were raised as a key barrier to adoption: “*The stairs are narrow anyway and to think of an extra handrail being put in, it would really narrow the staircase and that would feel more unsafe than it not being there*” (P6). Conversely, one participant stressed that, if a staircase is wide, holding onto handrails on both sides may not be possible. Participants also expressed differing preferences for handrail shape, with some preferring traditional contoured handrails for aesthetic reasons and others desiring a shape that provides additional grip. The lack of independent decision-making power was raised as a barrier. P6 highlighted: “*A lot of this is assuming […..] you have complete control over those decisions. Whereas you might live in a multi-generational household with different needs*”. Older adults held concerns about stigmatisation: “*that means I’m admitting I’m getting older, I’m getting more vulnerable. If new houses were built with two rails and it gradually became standard, it would help to overcome the perception that two rails means old and disabled. So I think I would start with trying to make it fashionable*” (P8). Overall group consensus indicated that an additional handrail is an acceptable intervention to older adults.

Professionals also identified handrails as an essential safety feature for older adults and acknowledged potential benefits of installing an additional handrail on home staircases. While professionals believed that most older adults would find handrails an acceptable intervention, potential implementation challenges were identified. Consistent with older adults, space constraints that might narrow the walking space were highlighted as the primary barrier to adoption of a second handrail. Moreover, the integrity and construction of walls were concerns and barriers for fitting handrails because “*Oftentimes the problem is the wall is not fit for a handrail*” (S10), and “*you don’t want to go through the [vapour] barrier*”. The height and shape of handrails were identified as important factors in determining effectiveness. “*What you’ll find with most of the traditional handrails is you can only get a grip over the top. If you do start to fall, that’s not going to prevent it at all. So it’s great for supporting your arm but it’s no good in terms of graspability*” (S1). Professionals were unanimously supportive of handrails as an intervention but highlighted the need for additional research evidence to determine the most effective handrail design and placement for preventing falls in different home settings. Group consensus was that an additional handrail is a high priority for implementation.

3. Ambient Lighting: All older adult participants agreed that adequate levels of light on stairs were important for safety. They felt this was particularly required to illuminate obstacles, especially for those with pets. A key barrier to modifying existing lighting was not understanding what type of lighting would be optimal. P8 stated, “*If you are in a lighting shop you can see millions of different light fittings, but knowing what would be really good to get from the stair safety point of view is another matter*”. A further barrier identified was that older adults may require assistance to move light fittings, which would add to the cost of making the change. The need for guidance on the most appropriate type and positioning of lighting, as well as where to access such guidance, were identified as facilitators to the adoption of this intervention. Group consensus was that ambient lighting is an acceptable intervention for older adults.

Professionals agreed on the importance of proper staircase lighting but stressed the need for more research evidence on the use of ambient lighting as a stair safety intervention. While some professional participants indicated an awareness of experimental studies, it was suggested that more comprehensive studies are required to understand how different lighting strategies and modifications affect stair use and fall risk for a diverse range of older adults. Even if effective lighting strategies can be evidenced in the lab, it was suggested that implementation in real-home settings can be challenging due to the habitual behaviour of end-users and economic cost: “*It comes down to behaviour*” (S8). In particular, the use of ambient lighting at night was discussed: “*You’ve got a generation that don’t want to leave lights on. They’re unplugging everything before they go to bed because that that’s the way they were brought up*” (S1). There were suggestions to consider researching the use of economical LED lighting and movement sensors to combat these issues. Group consensus was that ambient lighting would be a high implementation priority for professionals.

4. Changing stair coverings: Older adults were not aware that patterned stair coverings were a fall risk before the research team presented details of the intervention to them. P7 stated, “*I think changing the stair covering is probably only going to be something that you would do if you have had a fall, because you wouldn’t actually think about it prior to that*”. In addition to a lack of awareness, barriers to adoption raised by older adults included the economic cost and disruption of fitting new stair coverings. Once older adults were aware of the risk and the intervention, they suggested that everyone should be encouraged to consider the risk posed by stair décor upon the selection and purchase of a new stair covering or carpet. Education and awareness raising were highlighted as facilitators to adoption, which could be supported by carpet manufacturers and suppliers. Group consensus suggests that changing stair coverings to plain décor would be an acceptable intervention to older adults.

Professionals perceived that older adults would be resistant to making physical and aesthetic changes to their homes, even if safety risks are identified. Based on experience with patients in the community, S10 stated, “*If there’s a rug in the kitchen and it is a fall risk, they would not even move that. I’ve moved rugs myself for patients and the next time you visit it’s there again*”. Economic constraints for older adults and the healthcare sector were also cited as a barrier. Health professionals agreed that they would be willing to advise older adults about potential safety risks posed by patterned stair coverings but would not necessarily recommend the intervention as a top priority: “*For a lot of patients we see the majority of the falls can be multifactorial. We might identify the carpet as one of the things you might want to fix amongst a whole host of other things. But I think the reality of getting someone to change the carpet sort of becomes the last thing that you might try and do. You’d make it a lower priority*” (S5). Changing stair coverings to plain décor was agreed to be a moderate implementation priority to professionals.

5. Optical illusions on inconsistent steps: Older adult participants were unaware that inconsistencies in step dimensions may exist in home staircases and can present a significant fall risk. Barriers to adoption expressed by older adults related to doubts about efficacy (not understanding how it works), concern about impact on individuals with vision problems (if it would increase trip risk), and practical concerns about the ability to apply an optical illusion on carpeted stairs. However, participants expressed willingness to consider the intervention even if it results in aesthetic variance to the staircase. Suggested facilitators to adoption included the ability to adapt the intervention to be used on carpet stairs in keeping with personal home décor preferences and seeing a practical demonstration of the illusion in a real-life environment to help end-users better understand its efficacy. Group consensus suggests that an optical illusion would probably be an acceptable intervention for older adults.

Similarly, professionals raised concerns about the illusion’s mode of attachment and questioned if it would create an additional hazard if not securely placed. Health professionals were concerned that an illusion could increase confusion for older adults with cognitive impairment. While professionals recognised the applicability and potential of optical illusions as an intervention for ascending stairs, it was stressed that most falls happen on stair descent. S1 noted, “*This is good for going up, but going down you can’t see the illusion, so it doesn’t help you at all*”. Professionals emphasised end-user aesthetic preferences as a key barrier: “*People are really proud of their carpets. […..] unless somebody’s had the experience of falling they wouldn’t accept it aesthetically*” (S10). It was queried whether a learning effect would reduce long-term effectiveness. For home visitors who are unfamiliar with the stairs and older adults who climb stairs slowly, the intervention was considered potentially more beneficial. There were contrasting views on practical implementation: S3 stated: “*I quite like the idea because it’s so simple and it could be built into a service delivery plan relatively easily*”, while S5 indicated “*I think it’s a really good idea, but I’m not sure how the problematic step would ever be identified*”. As facilitators to adoption, professionals emphasised that further research in real-world home settings is required to establish potential risks and benefits for both ascent and descent. S3 stated: “*There is a liability issue around doing it if there is not proven evidence behind it*”. Group consensus was that optical illusions would be a moderate priority for implementation.

6. Physical edge highlighters: Older adults showed a lack of understanding of the intervention and expressed concern about efficacy, aesthetic preferences, and applicability on carpeted stairs. Some participants were concerned that the intervention would cause distraction: “*I don’t know whether that would make me clumsier somehow because of looking at the highlighters. I’d be thinking ‘am I getting them right’, as opposed to forgetting about it and just automatically walking upstairs*” (P7). Ensuring edge highlighters are designed to be flush to the step surface was suggested as a facilitator to adoption. A dislike of the aesthetic appearance was identified as a barrier and played a significant role in determining acceptability to older adults. Some participants indicated that they would consider edge highlighters if they could be seamlessly integrated within a carpeted surface or if the design could be ‘modernised’ to be in keeping with home decor. However, not all participants agreed: “*Whatever surface it’s on, I don’t think it would work for me*” (P3), “*I just don’t think it would be accepted by many people*” (P4). Group consensus suggested that physical edge highlighters would probably not be acceptable as an intervention in older adults’ homes.

Key barriers raised by professionals were consistent with older adults and related to application on carpets and aesthetical concerns of end-users. S1 stated, “*The big problem is people don’t want to look needy…or like you’re institutionalised*”. Professionals indicated that physical step edge highlighters may be more acceptable on external stairs, such as leading to people’s homes or in common areas of multi-occupied properties, where carpets are not used, and the aesthetic concerns of end-users have less impact. Like older adults, some professionals indicated concern about a physical strip edge highlighter posing an increased trip risk if it becomes loose or is not flush to the step. Painting on a strip edge was suggested as an alternative option that may be considered more feasible. Professionals emphasised that the feasibility of making modifications to the staircase would vary based on the level of control that older adults have over their home setting, with those living in rented or shared accommodation having less control over physical adaption decisions. Professional consensus held that physical edge highlighters would be a moderate implementation priority.

7. Lighting edge highlighters: Older adults were sceptical regarding the efficacy of lighting edge highlighters on home stairs, underpinned by confusion as to how they differed from ambient lighting. On being presented with an image of the intervention by the researchers, participants expressed dissatisfaction: “*That’s thrown me somehow. I don’t know, I don’t like the visual look*” (P7). Older adults were concerned that lighting edge highlighters could cause visual distractions and diversion of attention: “*When you first described it, I thought that would be a really good idea. But seeing it like that image, no, you’ll be looking down at it*” (P3). Some participants could see the benefits of lighting to highlight step edges, but individual preferences for the positioning and brightness of lighting varied. A practical demonstration of the intervention in a home setting to increase understanding was identified as a facilitator to adoption. Participants concluded that lighting edge highlighters were not acceptable as a stair safety intervention that older adults would adopt in their homes and preferred to improve ambient lighting.

In contrast to older adults, professionals saw potential for lighting edge highlighters to be beneficial to end-users and a viable intervention to implement in homes. The intervention was perceived by professionals to be more acceptable and easier to implement compared to other physical changes to the staircase (including physical edge highlighters, optical illusions, changing stair coverings, and rebuilding stairs). Professionals considered this intervention more practical because it could be presented to end-users as a ‘trial option’: “*I think this is a much easier sell because you can say we’re going to trial this, and it can potentially be removed if they don’t like it*” (S7). This flexibility was seen as a facilitator that could encourage implementation. Professionals emphasised the need to ensure that lighting fixtures do not protrude and are flush with the steps, to avoid creating trip hazards. Professionals suggested that further experimental research is required to test different lighting conditions and colours to find the most effective contrast for step detection. Group consensus was that lighting edge highlighters would be a moderate implementation priority.

8. Physical Training: Older adult participants agreed that physical training could be beneficial to prevent stair falls, particularly to address balance problems. Older adults identified the need for personalised exercise prescription depending on health status, recognising that some older adults may be limited in their ability to engage in exercise, while others may consider it unnecessary or already engage in regular physical activity (e.g., walking, cycling, and gardening). P5 stressed, “*For me that’s totally boring. I would never do it because I do other things, I keep fit and I eat healthily*”. Participants highlighted facilities and supervision as facilitators: P8 stated: “*who’s going to be supervising it and how you access it. For example, is it a good instructor who will tell you what you’re doing wrong, or one that just does an exercise and lets you follow them? I prefer having somebody who will actually give you some guidance*”. Preferences for the mode of delivery (e.g., group-based in person or individual home-based) differed between genders. Female participants preferred the in-person group activity, offering social interaction and motivation to engage, while male participants preferred individual home-based exercise programmes, because they perceived that “*Men are not group people. Even in gyms or classes, 99% are women. Fellas tend to stay on their own. I don’t know why*” (P2). Group consensus suggests that physical training would be an acceptable intervention for older adults.

Health professionals emphasised that strength and balance exercises improve the capability and confidence of older adults in relation to falls and stair use and highlighted that home exercise plans are generally acceptable to older adults. Professionals noted that existing research evidence on physical training shows benefits for general falls but suggested that the impact on serious stair falls may be more limited. They also questioned whether there could be unintended consequences resulting from increased confidence (e.g., increased stair use, potentially becoming less cautious, leading to more opportunities for falls). Despite this, professionals agreed on the value of physical exercise for improving confidence and stair usability, even if they may not directly reduce the risk of serious falls. Consensus was that physical training would be a moderate implementation priority.

9. Education: Older adult participants were in agreement with P7: “I think it’s [education about stair safety] good for everybody because otherwise, until it happens, you don’t really think about it”. Older adults suggested that educational programmes should be proactive in highlighting potential stair safety issues before serious falls occur. As a facilitator to adoption, there was consensus that educational interventions would be more acceptable to older adults if signposted or delivered by professionals in the health sector. The main barrier to adoption was related to differing preferences for the mode of delivery. Female participants indicated a preference for group activities, with one stating the following: “One of the problems with things like leaflets or emails is that it doesn’t promote a conversation…[but] somebody coming and giving you a talk is it actually promotes a conversation. Using community groups you can start conversations” (P8). In contrast, male participants were less inclined to join group-based interventions and preferred accessing information individually and/or remotely (e.g., online resources, phone app). Having a range of delivery options was expressed as a facilitator to adoption. Group consensus suggests that education is an acceptable intervention for older adults and that education on stair falls should be expanded to the general public to raise wider awareness and promote social encouragement for older adults.

Professionals were generally positive about using education as an intervention. Professionals agreed that there could be education on important stair safety principles, not only to older adults but ‘to the masses’ as a facilitator to adoption: “*The biggest resource is actually the relatives of the older people. We need to build their knowledge*” (S8). Professional consensus was that an educational intervention would be a high implementation priority.

10. Skill Training: Older adult participants generally agreed that skill training on the safe use of home stairs would be helpful because “*I had a fall on stairs […..] but I know I could have done something to avoid it*” (P6). Gaining access to skill training and motivating older adults, particularly non-fallers, were barriers to adoption. “*People who have had falls will be more motivated probably. But practically I don’t know how you’d get it across to the general public*” (P8). Participants suggested that skill training could be delivered in a class-like setting, such as at a fitness centre or a community centre. Consensus was that skill training is considered an acceptable intervention for older adults.

Professionals were optimistic about a skill training intervention. However, participants suggested that it would need to be personalised and specific, or transferable, to the end-users’ own home setting and account for individual ways of navigating stairs to facilitate adoption. Professionals stressed that practising safe stair use on generic stairs (e.g., in a hospital setting) may not be realistic or translate to improved stair use in end-users’ own homes. Tailored strategies for negotiating individual home stairs, working with existing capabilities and preferences, were therefore considered essential for maintaining engagement and successful implementation. S3 stated, “*My experience working with older people is they’re incredibly proud and independent. If something’s not tailored for their circumstance they don’t really see the relevance of it*”. However, resource constraints in healthcare settings (such as the National Health Service) were acknowledged as a key barrier in providing tailored/individualised training interventions. Professional consensus deemed skill training to be a high priority for implementation.

#### 3.2.3. Summary of Focus Group Results

The focus group findings suggest that there are multiple factors that may support (facilitators) or hinder (barriers) older adults’ and professionals’ acceptance and adoption or implementation of stair-fall prevention interventions in real-world settings, with key themes summarised in Table 4. Figure 3 summarises older adults’ consensus ratings towards the acceptability of the 10 stair-fall prevention interventions discussed, compared to professionals’ consensus ratings towards implementation priority for the interventions.

## 4. Discussion

This is the first study to establish older adult and professional stakeholder perspectives on stair-fall prevention interventions. Some of the findings are consistent with the previous literature on general falls which suggests older adults see fall prevention as relevant to others but often do not acknowledge their personal fall risk [31,38,50] and may reject the idea that they need fall prevention advice/help because they see themselves as fit and healthy [31,50]. However, while studies show that older adults attribute general falls to external causes [30,31], we found that older adults surveyed perceived personal behaviour as the highest risk factor for home stair falls, and almost half of the participants who had experienced a stair fall cited their ‘own behaviour’ as the cause. In contrast to studies suggesting that older adults believe falls are an inevitable consequence of ageing [8,31], most older adults surveyed believed stair falls are preventable. Whilst older adults surveyed expressed high interest in stair-fall prevention generally, they demonstrated comparatively less interest in using specific interventions to prevent stair falls. In part, this could be due to a lack of awareness, given that almost half of the survey participants identified better understanding of the benefits of stair-fall prevention and of intervention options as facilitators to adoption. The focus groups allowed stakeholder attitudes toward specific stair-fall prevention interventions to be explored in more depth. The key themes emerging from the focus group discussions (summarised in Table 4 and Figure 3) are discussed in relation to the existing literature in Section 4.1, Section 4.2 and Section 4.3.

### 4.1. Barriers

Common barriers across all interventions related to a lack of awareness and understanding (of stair-fall risk or the intervention), perceived personal relevance depending on individual health status, and limitations of research evidence. Such barriers align with the previous literature on older adults’ attitudes towards general fall prevention. Previous studies suggest that older adults advocate fall prevention changes for others but often under-estimate their personal fall risk [31,38,50], which could lead to a lack of interest or resistance to accepting stair-fall prevention interventions. Comparable with previous studies [50,51], older adults who considered themselves to be fit and healthy or already active felt that certain stair-fall prevention interventions (e.g., physical training, skill training) were acceptable but not personally relevant to them. Meanwhile, for older adults with health issues (e.g., cognitive impairment, poor vision), certain interventions were considered unsuitable or potentially adding to fall risk or confusion (e.g., optical illusions, edge highlighters). This emphasises the need for high-quality and individual-specific evidence of intervention effectiveness, which the professionals highlighted as a requisite for recommendation and implementation.

Common barriers across home environment interventions included cost, practicalities (e.g., finding reliable tradespersons, guidance on effective changes), resistance to aesthetic changes and home disruption, stigma, and lack of control. Other studies suggest that older adults are often resistant to accepting home modifications to prevent falls [8,14,36,38]. Aesthetics and potential stigma (e.g., being viewed as vulnerable or institutionalised) associated with fall prevention interventions are commonly reported reasons that may contribute to such resistance [14,31,36,38]. Stakeholder research on home adaptations to support later life [52] similarly suggests that older adults may be reluctant to accept adaptations or delay installation due to product aesthetics (e.g., clinical appearance), negative associations, and financial constraints. Our study confirms that such factors may also act as barriers to the adoption of home environment stair-fall prevention interventions. The lack of decision-making power or control over physical changes to the home was emphasised as a barrier for older adults living with others or renting. Likewise, Tural et al. [14] found that living with others negatively impacts older adults’ attitude toward the use of stair mobility products, and Kruse et al. [36] suggest that older adults’ sense of control over their home is a key factor in accepting home modifications to prevent falls. In contrast to Kruse et al. [36], our findings did not suggest that older adults were uninterested or saw no benefit from home modifications to prevent stair falls. However, their interest and acceptance of modifications were influenced by their awareness and understanding.

While behavioural and personal capability interventions were generally highly acceptable to focus group participants, key barriers across such interventions related to a lack of resources in the health sector (NHS) to offer tailored support and maintain older adults’ engagement. Issues around engagement and adherence are common barriers reported in studies on older adults’ attitudes towards fall prevention exercise programmes [8,53].

### 4.2. Facilitators

Common facilitators across all interventions were related to increasing awareness and knowledge of stair-fall risks and intervention options, accessing advice or guidance about appropriate interventions, promoting social support and encouragement, and tailored/flexible options to meet diverse needs and preferences. It was recognised that older adults often only consider or accept interventions once something goes wrong (e.g., after a stair fall), particularly with regard to modifications to the home environment. The literature on home modifications to support ageing in place similarly highlights that they are often done following a ‘trigger’ [52]. Therefore, in keeping with World Falls Prevention Guidelines [1], proactively raising awareness specifically for stair falls and prevention options was a prominent facilitator for most interventions investigated. Social encouragement and support (e.g., from friends, family, and health professionals) was found to be a facilitator to ‘normalise’ interventions and encourage adoption, which is congruent with other studies [29,31,34]. Therefore, the importance of raising awareness of stair falls and prevention in the general population, not just in older adults, emerged as a strategy to reduce the stigma that older adults associate with stair-fall prevention interventions. This may include peer role models to share experiences of the benefits of successful adoption of interventions. Across all interventions, participants highlighted the need for personalised/tailored interventions to meet individual needs and preferences, according to socio-demographic characteristics or the home environment. This is consistent with studies of older adults’ and health practitioners’ perspectives on general fall prevention programmes [29,30,31,32,54]. Consistent with research on later life home adaptations [52], although older adults expressed dislike for the aesthetic appearance of certain environment interventions, this did not mean that they were unacceptable if the benefits of the intervention were clearly defined. In some instances, professionals were more pessimistic than older adults about the acceptability of interventions that result in aesthetic changes to the home. This contrast highlights the importance of efforts to provide understanding, as well as practical support, to older adults when recommending home environment interventions.

There was evidence that intervention safety and effectiveness were important facilitators for both older adults and professionals. Participants expressed particular concern about lesser-known or novel environment interventions, such as optical illusions and step edge highlighters. To address a lack of understanding and/or to evidence efficacy, the use of practical demonstrations in a home setting was suggested by older adults and professionals. Importantly, professionals desired experimental research evidence to support their decisions and practical implementation. Additionally, our findings suggest that home environment interventions that are customisable to meet end-user aesthetic preferences (e.g., ability to use edge highlighters and optical illusions on carpet) and are socially acceptable (e.g., two handrails) are more likely to be acceptable to older adults. It was suggested that tackling stigma associated with home environment interventions could be achieved in part by designing new build homes with improved stair safety standards (e.g., safer step dimensions and two handrails).

While behavioural interventions (education and skill training) were considered highly acceptable, providing this information to the target audience and ensuring that intervention options are accessible to older adults was identified as critical for encouraging adoption. Consistent with Khong et al. [55] and Yardley et al. [38], older adults generally preferred that stair-fall prevention information would be disseminated or referred to by health professionals. Older adults also suggested that community groups could be used to raise awareness and encourage conversation (e.g., share experiences). In line with the literature regarding engagement with fall prevention activities [28,56,57], the opportunity for social interaction was identified as a facilitator to behavioural and capability interventions. However, preferences differed by gender, with female participants expressing a greater preference for group and in-person interventions, while male participants indicated preference for individual and remote options (e.g., home-based, online sources, and smartphone applications). Similarly, Yardley et al. [38] found that women were more likely to attend group fall prevention exercise training. Dorresteijn et al. [54] identified that background characteristics, including gender, are associated with preferences for specific fall-prevention programme formats. While past research suggests adults over age 75 are less likely to own digital devices and use the internet to obtain health information [58], findings from the present study indicate that older adults view online sources and smartphone apps as viable tools for home-based physical training and education interventions. This may reflect a generational shift in technology adoption and engagement over time. Concurring with other studies [35,37,38,54,55], a variety of delivery formats, including group and home-based, to meet diverse needs and preferences (e.g., different genders, age groups, health status, etc.), is a key facilitator to encourage the adoption of behavioural and capability interventions.

Given the frequency that cost/resource constraints were mentioned, financial support for older adults and/or funding for healthcare and housing providers was a commonly cited facilitator, particularly to assist with environmental interventions. Considering the substantial healthcare costs associated with falls, investing in fall prevention could be a cost-effective approach in the long term [28,59]. There is evidence in the UK that minor home adaptations to prevent (non-specific) falls can be cost-effective [60,61].

### 4.3. Intervention Acceptability and Priority

Based on group consensus, Figure 3 demonstrates that older adults’ and professionals’ held similar perceptions with regard to behavioural interventions (education and skill training), additional handrails, and ambient lighting. Across both focus groups, these interventions were perceived to be highly acceptable/high priority. There was also consensus between both older adults and professionals that rebuilding stairs as a means of stair-fall prevention had low acceptability/low priority. Physical training and changing stair coverings to plain décor were generally acceptable to older adults but perceived with slightly lower implementation priority by professionals. There was greater divergence in the perceptions of older adults and professionals towards other interventions that require physical changes to the home environment, including step edge highlighters, lighting edge highlighters, and optical illusions. Edge highlighters were less acceptable to older adults than to professionals. The greatest diversity in perceptions was with regard to lighting edge highlighters, considered the least (not) acceptable intervention to older adults, but viewed with more promise by professionals. In general, older adults in the focus group demonstrated higher interest in and acceptance of stair-fall prevention interventions following an explanation about the interventions, compared to older adults surveyed where intervention options were simply listed. This supports the need for awareness raising and greater understanding of intervention options by older adults, which were also highlighted as the main facilitators to adoption in the survey.

### 4.4. Limitations and Future Directions

The findings should be considered in light of this study’s limitations. The data reflect participants’ self-reported attitudes towards adopting stair-fall prevention interventions and not their actual behaviour. Some research with older adults suggests that, despite positive attitudes towards an intervention or product, intention to use can be low [14]. Our study had a small focus group sample size with low representation from older adult males (25%). Other studies involving older adults [38,62,63] also find higher participation rates by female participants than male participants (e.g., female participation rates ranged between 72 and 83%). Survey and focus group participants had little diversity in terms of ethnic background and geographical scope; thus, the generalisability of results to different cultures may be limited. The primary use of online methods to recruit older adult participants may have excluded digitally disconnected individuals, potentially introducing sample bias. However, the participants capture a diversity of older age groups, living arrangement/housing type, health status, and fall history, as well as professionals representing both health and housing sectors. There is consistency with the previous fall literature regarding prominent barriers and facilitators to the adoption of stair-fall prevention interventions, as well as new insights which provide directions for future work. Future research should explore effective delivery formats for behavioural interventions (education, skill training), the impact of gender on attitudes towards interventions and perceptions on the use of digital technology for fall prevention (e.g., smartphone apps, sensors). More empirical laboratory research is required to determine optimal lighting conditions for stair safety, the application of edge highlighters and optical illusions on carpeted surfaces, and the use of optical illusions on stair descent. Cost–benefit analysis of implementing different stair-fall prevention interventions could be explored to support the development of funding assistance programmes for the most cost-effective interventions.

## 5. Conclusions

Prevention interventions to reduce stair falls in older adults are complex and multifaceted. Essential to designing and effectively implementing such interventions is an understanding of the attitudes and perceptions of key stakeholders. To our knowledge, this is the first study to investigate stakeholder attitudes, including older adults and professionals, towards a range of stair-fall prevention interventions for community-dwelling older adults in the UK. This study considered perceptions towards ten stair-fall prevention interventions, including interventions involving changes to personal behaviour and capability and changes to the home environment. The findings contribute to understanding older adult and professional perceptions on the acceptability of stair-fall prevention interventions and potential barriers and facilitators to their adoption in real home settings. The findings will be valuable for a range of stakeholders including policy makers, health and housing practitioners, educators, and researchers. Understanding stakeholder perceptions will guide future research aimed at preventing stair falls in older people. It will help health and housing professionals and researchers in the design of stair-fall prevention interventions and facilitate expanding the scope for their implementation and adoption by end-users in real home and community settings.

## Figures and Tables

**Table 1:** Survey participant characteristics.

Question	Response Options	Percent of Respondents
Age	55–59	21%
	60–64	27%
	65–69	24%
	70–74	10%
	75–79	8%
	80–84	7%
	85–89	2%
	90+	1%
Gender	Male	45%
	Female	55%
Ethnicity	White	81%
	Black/African/Caribbean/Black British	7%
	Asian/Asian British	7%
	Mixed/Multiple Ethnic Groups	2%
	Other Ethnic Group	2%
	Undisclosed	1%
Living alone	Yes	31%
	No	69%
Property type	Detached house	26%
	Semi-detached	25%
	Terraced house	10%
	Bungalow (containing stairs)	21%
	Flat/Apartment (containing/accessed via stairs)	18%
Housing tenure	Owner occupied	80%
	Private rented	13%
	Social rented	6%
	Other	1%
Previous stair fall	No	44%
	Yes, one fall	32%
	Yes, more than one fall	24%

**Table 2:** Older-adult focus group participant characteristics.

Participant ID	Age	Gender	Ethnic Group	Stair-Fall History	Health Impairment	Living Alone	House Type
P1	70–74	Male	White	Non-faller	Mobility, Dexterity	No	Semi-detached
P2	70–74	Male	White	Non-faller	None	No	Detached
P3	75–79	Female	White	Non-faller	Hearing	Yes	Terrace
P4	80–84	Female	White	Non-faller	Other (not specified)	Yes	Semi-detached
P5	75–79	Female	White	Non-faller	None	Yes	Terrace
P6	65–69	Female	Other Ethnic Group	Faller	Hearing, Mobility	Yes	Terrace
P7	60–64	Female	White	Faller	Vision	No	Detached
P8	70–74	Female	White	Faller	None	Yes	Detached

**Table 3:** Professional stakeholder adult focus group participant characteristics.

Participant ID	Professional Role
S1	Housing and Health and Stair Safety Consultant
S2	Stair Safety Specialist
S3	Housing and Falls Expert
S4	Surveyor for Social Housing Provider
S5	Consultant Geriatrician
S6	Consultant Geriatrician, NHS
S7	Lead Practitioner for Prevention of Falls (Inpatient)
S8	Clinical Fall Lead and Pathway Co-ordinator (Outpatient)
S9	Community Physiotherapist
S10	Occupational Therapist, NHS
S11	Specialist Falls Nurse, NHS

**Table 4:** Barrier and facilitator themes to the adoption of stair-fall prevention interventions.

Intervention		Barrier Themes	Facilitator Themes
Environment	Rebuild stairs Optical illusion Change stair coverings (plain décor) Physical edge highlighter Lighting edge highlighter	Cost	Financial assistance/funding
		Space constraints	Guidance and advice
		Disruption/rehousing	Raising awareness
		Lack of decision-making power or living with others	Practical demonstrations
		Not knowing where to find reliable advice/tradesmen	Research evidence
		Lack of awareness of risk	Adaptable design to meet aesthetic preferences
		Doubts about efficacy/Lack of understanding of intervention	Social support and encouragement
		Suitability for individual health status	
		Aesthetics	
		Stigma and pride	
	Ambient lighting Additional handrail	Space constraints	
		Cost	Economical options
		Lack of understanding (e.g., safest changes, accessing tradesmen)	Guidance and advice
		Lack of decision-making power or living with others	Raising awareness
		Stigma and pride	Social support and encouragement
		Habitual behaviour	Behaviour change
Behaviour and Capability	Physical training Skill training Education	Relevance/suitability for individual health status	Tailored to individual needs and circumstances
		Lack of awareness of risks	Raising awareness in general public
		Access by and promotion of information to target audience	Trusted referrals and advice
		Personal preferences for delivery style	Variety of delivery options
		Motivation/maintaining engagement of end-users	Social support and encouragement
		Lack of resources in health sector	

## Data Availability

Data are contained within this article.

## Appendix A. Copy of Older Adult Survey Questions

Background questions

1. What is your age?

◻ 55–59 ◻ 60–64 ◻ 65–69 ◻ 70–74 ◻ 75–79 ◻ 80–84 ◻ 85–89 ◻ 90+

2. What is your gender?

◻ Male ◻ Female ◻ Transgender ◻ Prefer not to say

3. What is your ethnic group?

◻ White ◻ Mixed/Multiple Ethnic Groups

◻ Asian/Asian British ◻ Other ethnic group

◻ Black/African/ Caribbean/Black British ◻ Prefer not to say

4. How is your overall health in general?

◻ Excellent ◻ Good ◻ Fair ◻ Poor

5. Do you have any conditions or illnesses that affect you in any of the following areas? Select any that apply

◻ Vision (e.g., blindness or partial sight)

◻ Hearing (e.g., deafness or partial hearing)

◻ Mobility (e.g., walking short distances or climbing stairs)

◻ Dexterity (e.g., lifting and carrying objects, using a keyboard)

◻ Learning or understanding or concentrating

◻ Memory

◻ Mental Health

◻ Stamina or breathing or fatigue

◻ Socially or behaviourally (e.g., autism spectrum disorder (ASD), Asperger’s, ADHD)

◻ Other (please specify..............................................................................................)

6. How would you describe your activity status?

◻ Very active ◻ Somewhat active ◻ Somewhat inactive ◻ Mostly inactive

7. What type of property do you live in?

◻ Detached house ◻ Bungalow (containing stairs)

◻ Semi-detached house ◻ Flat/Apartment (containing or accessed via stairs)

◻ Terraced house

◻ Other (please specify) ………………..............................................

8. Do you own or rent your current home?

◻ Owner occupied ◻ Private rented ◻ Social rented ◻ Other (please specify.......................................................................................................)

9. Are you currently living alone?

◻ Yes ◻ No

Awareness of stair falls and causes of falls

10. Please indicate the extent that you agree or disagree with the statements in the table below:

	Strongly disagree	Disagree	Agree	Strongly agree	Undecided
‘Falling on stairs is a concern for older adults’	◻	◻	◻	◻	◻
‘Stair falls are common for older adults’	◻	◻	◻	◻	◻
‘Stair falls in older adults are preventable’	◻	◻	◻	◻	◻

11. Rate the extent that you think each of the factors in the table below are a risk factor for stair falls:

	No risk	Low risk	Moderate risk	High risk
Medical factors (e.g., healthcondition, disability, impact of medication)	◻	◻	◻	◻
Balance and mobility problems	◻	◻	◻	◻
Visual or sensory problems	◻	◻	◻	◻
Level of fitness/inactivity	◻	◻	◻	◻
Psychological factors (e.g., anxiety, fear of falling)	◻	◻	◻	◻
Environmental factors (e.g., stair design, stair covering, presence of handrail, lighting, obstacles)	◻	◻	◻	◻
Personal behaviour (e.g., rushed movement, distracted attention, carrying items, alcohol consumption)	◻	◻	◻	◻
Demographic factors (e.g., living alone)	◻	◻	◻	◻

Personal stair-fall risk and experience of falls

12. Please indicate the extent that you agree or disagree with the statements in following questions:

	Strongly disagree	Disagree	Agree	Strongly agree	Undecided
‘I consider myself at risk of falling on stairs at home’	◻	◻	◻	◻	◻
‘I am fit and healthy so I don’t see myself at risk falling on stairs’	◻	◻	◻	◻	◻

13. Have you ever had a fall on stairs in your home?

◻ Yes, once ◻ Yes, more than once ◻ No

If you selected ‘Yes’, why do you think you have fallen on stairs (what caused you to fall)?

◻ Because of your behaviour on the stairs (e.g., rushing, carrying objects, talking while moving, foot placement)

◻ Because of your health status/a medical condition

◻ Because of the staircase/the environment

◻ A combination of the above factors

◻Other—please specify....................................................................

14. Do you consider your stairs at home to be safe (not presenting a fall risk)?

◻ Yes ◻ No ◻ Don’t Know

If you said ‘No’/‘Don’t know’, what aspect(s) of your stairs do you deem to be unsafe? Select any that apply

◻ Steep stairs/Stair dimensions (tall riser and/or short tread)

◻ Absence of handrail

◻ Slip resistance/type of surface covering

◻ Narrow staircase

◻ Handrail in disrepair or loose

◻ Handrail not set at appropriate height

◻ Gaps between stairs (open risers)

◻ Stairs (steps or covering) in disrepair

◻ Poor lighting/visual condition

◻ Décor (e.g., busy patterns on carpets)

◻ Cluttered environment

◻ Other—please specify ....................................

15. Please indicate the extent that you agree or disagree with the statements in the table below:

	Strongly disagree	Disagree	Agree	Strongly Agree	Undecided
‘I consider stair fall prevention at home a priority’	◻	◻	◻	◻	◻
‘I am aware of what changes can be made at home to reduce the risk of stair falls’	◻	◻	◻	◻	◻

16. Would you be interested in making changes to your home environment to reduce risk of stair falls?

◻ Yes ◻ Possibly ◻ No

◻ I have already made changes

17. Would you be interested in engaging in behavioural changes to reduce risk of stair falls?

◻ Yes ◻ Possibly ◻ No

◻ I have already made changes

18. Which of the following do you think would be good adaptions or interventions to improve hoe stair safety? Select any that apply

◻ Changing handrail to be at an appropriate height

◻ Additional handrail

◻ Slip resistant stair covering

◻ Clearly marked edges on stairs

◻ Plain décor on steps (e.g., replacing a carpet that has busy patterns)

◻ Changes to staircase lighting (artificial or natural light)

◻ Doing targeted exercises to improve balance or muscle strength

◻ A stair lift

19. Is there anything that would encourage you to adopt interventions or adaptions that reduce risk of stair falls in your home? Select any that apply

◻ Having a better understanding of stair fall risks and implications of falls

◻ Having a better understanding of what interventions or adaptations are available in my home

◻ Having a better understanding of the potential benefits of stair falls-prevention interventions

◻ Hearing about stair falls-prevention interventions from others who have used them

◻ Being referred by a healthcare professional

◻ Social support (e.g., encouraged by friends/family/carer)

◻ Financial support to make changes

◻ Other—please specify…………………………………………….

20. Is there anything that stops or is delaying you from adopting interventions or adaptions that reduce risk of stair falls in your home? *Select any that apply*

◻ Not knowing what adaptations or interventions might be possible

◻ Not knowing where to go to get reliable advice about suitable adaptions

◻ Needing assistance to make the adaptions or changes

◻ Cost or affordability concerns

◻ Concern about the appearance of physical changes to my home

◻ Not being able to get consent from a landlord to make physical changes

◻ Other person(s) in the home or family not wanting the adaptations or interventions

◻ Concern about being seen as vulnerable or other stigma

◻ I don’t think I need them yet

◻ Other—please specify……………………………………

Survey Complete

## Appendix B

**Table A1:** Stair-fall prevention interventions investigated.

Intervention	Category	Description of Intervention
Rebuilding stairs	Environment Intervention	Replacing existing staircase to meet UK building regulations specified in approved Document K (e.g., step rise, going and pitch).
Ambient lighting	Environment Intervention	Increasing illumination on stairs by adding more sources of ambient light, moving light fittings, and/or modifying sources of lightning. Intended to reduce fall risk by allowing a safer placement of feet on step tread (e.g., smaller foot overhang) and adequate foot clearance over step edge during stair ascent and descent.
Changing stair coverings (to plain décor)	Environment Intervention	Removing patterned carpets and coverings and replacing with plain, single colour step-surface decor to improve delineating step edges. Intended to reduce fall risk by allowing safer placement of feet on step tread mainly during stair descent.
Optical illusion on inconsistencies	Environment Intervention	Superimposing a horizontal–vertical illusion design on the riser of an inconsistently taller step within the staircase. Intended to improve foot clearance over the edge of an inconsistent step during stair ascent.
Edge highlighters (physical)	Environment Intervention	Superimposing along the edge of steps, physical strips, and nosings of adequate colour contrast and slip resistance. Intended to (1) improve the placement of feet with the available step tread during stair descent and (2) minimise the risk for a slip if there is a large foot overhang during stair descent.
Edge highlighters (lighting)	Environment Intervention	Adding appropriate lightning sources at specific places around the staircase to highlight the edge of the steps. Intended to reduce fall risk by allowing safer placement of feet on the step tread and adequate foot clearance over the step edge.
Additional handrail	Environment Intervention	Adding a second handrail of standards and characteristics specified in UK building regulations Document K. Intended to allow the safer placement of feet on step tread by improving balance control during stair descent.
Physical Training	Functional Capability Intervention	Improvement of relevant functional capabilities which deteriorate with old age, e.g., muscle strength and balance, through systematic engagement in appropriate exercise training. Intended to reduce fall risk by allowing a better controlled placement of feet on the step tread during stair ascent and descent and more successful recovery following a postural instability (e.g., grabbing onto handrails quickly and effectively following a collision with a step edge during stair ascent).
Education	Behavioural Intervention	Increasing awareness around factors influencing stair-fall risk and individual and socio-economic consequences of stair falls. Intended to improve safety by providing knowledge to enable end-users to implement effective and pragmatic measures to improve stair safety.
Skill Training	Behavioural Intervention	Improving stepping technique through training from relevant health practitioners (e.g., occupational therapists). Intended to reduce fall risk by teaching users how to move their feet on steps, taking into account individual functional limitations and actual home stairs and environmental design. Intended to improve safety by teaching tailor-made routines and practices on stairs, accounting for end-user circumstances.
